# Body size determines eyespot size and presence in coral reef fishes

**DOI:** 10.1002/ece3.6509

**Published:** 2020-07-11

**Authors:** Christopher R. Hemingson, Peter F. Cowman, David R. Bellwood

**Affiliations:** ^1^ College of Science and Engineering James Cook University Townsville Qld Australia; ^2^ Research Hub for Coral Reef Ecosystem Function James Cook University Townsville Qld Australia; ^3^ Centre of Excellence for Coral Reef Studies James Cook University Townsville Qld Australia

**Keywords:** body size, constraints, coral reef fishes, eyes, eyespots, ocellus

## Abstract

Numerous organisms display conspicuous eyespots. These eye‐like patterns have been shown to effectively reduce predation by either deflecting strikes away from nonvital organs or by intimidating potential predators. While investigated extensively in terrestrial systems, determining what factors shape eyespot form in colorful coral reef fishes remains less well known. Using a broadscale approach we ask: How does the size of the eyespot relate to the actual eye, and at what size during ontogeny are eyespots acquired or lost? We utilized publicly available images to generate a dataset of 167 eyespot‐bearing reef fish species. We measured multiple features relating to the size of the fish, its eye, and the size of its eyespot. In reef fishes, the area of the eyespot closely matches that of the real eye; however, the eyespots “pupil” is nearly four times larger than the real pupil. Eyespots appear at about 20 mm standard length. However, there is a marked decrease in the presence of eyespots in fishes above 48 mm standard length; a size which is tightly correlated with significant decreases in documented mortality rates. Above 75–85 mm, the cost of eyespots appears to outweigh their benefit. Our results identify a “size window” for eyespots in coral reef fishes, which suggests that eyespot use is strictly body size‐dependent within this group.

## Graphical abstract

Eyespots can help deter predation. We found that the eyespot's “pupil” is much larger than the real pupil. Furthermore, eyespots are less common in fishes as they become adults.

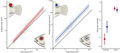

## 1 INTRODUCTION

**FIGURE 1:**
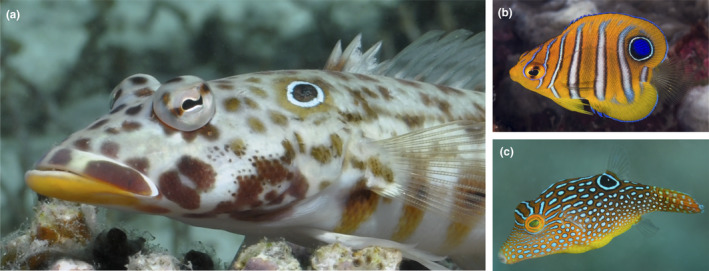
The diversity of coral reef fishes with eyespots. (a) A sandperch, *Parapercis clathrata*, (b) an angelfish, *Pygoplites diacanthus*, and (c) a pufferfish, *Canthigaster solandri*. Photographs with permission from Rick Stuart‐Smith (a) and François Libert (b,c)

Many organisms use coloration for differing fitness benefits (Cuthill et al., 2017). Quite often, coloration is tuned to aid in survival (Caro & Allen, 2017). One example may be the eyespot or ocellus, a highly conspicuous marking that is believed to resemble the eyes of some vertebrates (Blest, 1957). It is comprised of a dark, circular “pupil,” surrounded by a pale ring that contrasts against both the pupil and the base color of the organism. Eyespots are extremely common in nature and are found in numerous taxa from phylogenetically distinct lineages, including insects, molluscs, amphibians, crustaceans, birds, and fishes (Kodandaramaiah, 2011; Stevens & Ruxton, 2014). Furthermore, eyespots are found on species that have vastly different morphologies, life histories, behaviors, and colorations, suggesting a widespread underlying role (Figure 1; Marshall, Cortesi, de Busserolles, Siebeck, & Cheney, 2018).

The conspicuous nature of eyespots, along with their widespread occurrence, has made them historically appealing to study. Indeed, this marking has received continual research attention since the 19th century (Poulton, 1890). Independent studies on different taxa have identified multiple, different, functions of eyespots. These include mate selection and preference (Kodandaramaiah, 2011; Robertson & Monteiro, 2005), intraspecific competition between juveniles and adults (Gagliano, 2008; Gagliano & Depczynski, 2013), reproduction (Egger, Klaefiger, Theis, & Salzburger, 2011; Theis, Salzburger, & Egger, 2012), and anti‐predation benefits (Kjernsmo & Merilaita, 2013, 2017; Stevens, Stubbins, & Hardman, 2008).

While extensive in nature, the majority of research has focused on the latter topic, describing how eyespots promote survival through two, primary mechanisms: predator deflection and intimidation. (Lyytinen, Brakefield, & Mappes, 2003). Deflective eyespots operate by directing strikes away from vital organs, like the real eyes and head (Kjernsmo & Merilaita, 2013; Prudic, Stoehr, Wasik, & Monteiro, 2014). Alternatively, eyespots can function by intimidating potential predators by making the individual bearing them appear larger in size (De Bona, Valkonen, López‐Sepulcre, & Mappes, 2015). Most research has examined these mechanisms by using experimental approaches, primarily on insects (e.g., caterpillars, butterflies, and moths; Hossie & Sherratt, 2013; Lyytinen et al., 2003). Often, this entails presenting or manipulating the pattern on a prey item and subjecting it to a model predator species to record the interaction (De Bona et al., 2015; Prudic et al., 2014). These approaches have been particularly informative in describing how the pattern functions and which features may make it successful.

Although these experiments have been supported by field observations (Hossie & Sherratt, 2012, 2013) surprisingly few studies have utilized more broad, analytical approaches to describe commonalities in eyespots among many species (but see Ho, Schachat, Piel, & Monteiro, 2016; Hossie, Skelhorn, Breinholt, Kawahara, & Sherratt, 2015). Indeed, several key questions remain: for example, how does the size of the eyespot relate to the size of the real eye, if present? And at what size do eyespots become irrelevant (i.e., when do animals lose them)? These basic questions, albeit critically important, have received relatively little attention (cf. Hossie et al., 2015; Karplus & Algom, 1981). Body size and morphology often strongly constrain the life history traits of many organisms. (Bellwood & Choat, 1990; Berumen, Pratchett, & Goodman, 2011; Mihalitsis & Bellwood, 2017). Therefore, approaching eyespots from a more general perspective, and analyzing these questions across many species, may provide insights into what makes this marking so common and how it may function.

Coral reef fishes offer an ideal group to study this marking. Unlike insects that have a compound eye, reef fishes have an eye with a pupil and iris that the eyespot may resemble. This similarity allows for direct comparison between features of the eyespot and features of the real eye; a comparison not possible in insects. Furthermore, reef fish coloration is highly adaptive (Hemingson, Cowman, Hodge, & Bellwood, 2019) and many species gain or lose eyespots through development. To understand what rules shape the presence of eyespots in reef fishes we ask, (a) how does the eyespot compare to the eye in size and (b) at what body size are eyespots typically gained or lost in coral reef fishes? In doing so, we shed light on the processes that may shape eyespot use in one of the world's most diverse and colorful group of vertebrates.

## 2 MATERIALS AND METHODS

### 2.1 Defining an eyespot

For reef fishes, we defined an eyespot based on three criteria. (a) The entire eyespot needs to be approximately circular or elliptic in shape. (b) It has a dark (typically black) interior circle or ellipse that is surrounded by no less than 75% of its circumference by a concentric ring of differing, much lighter color (typically white). (c) There could be no more than 10 eyespots present on an individual. These criteria were chosen to ensure that the pattern is as a distinct marking that is visually conspicuous against rest of the fish's coloration. Strict criteria are necessary since there is a broad spectrum of markings present on coral reef fishes. Establishing these criteria allows us to focus on the species with a consistent eyespot form to determine what influences their presence and appearance.

### 2.2 Image sourcing and data collection

**FIGURE 2:**
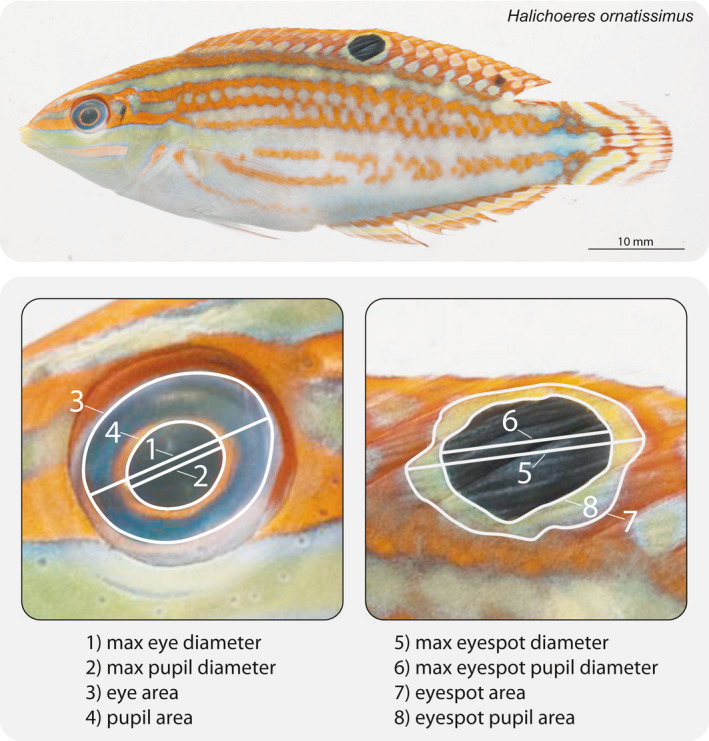
An example photograph from the Smithsonian Institute's Division of Fishes Collections. On top is the raw image of *Halichoeres ornatissimus*. The scale bar has been added. In this photograph, the fins are exposed, which permits morphological measurements of both the eye and, most importantly, the eyespot. In fishes with folded fins, only the presence, not the size, of the eyespot can be recorded. Below are the 8 measurements (both linear and area) of the eye and eyespot features. Photograph: 2006 Moorea Biocode/Jeffrey T. Williams, Smithsonian Institution

Bony fishes from four geographically distinct ecoregions were surveyed for the presence of eyespots (The Great Barrier Reef and Coral Sea, the Red Sea and Indian Ocean, the Caribbean Sea, and the Tropical Eastern Pacific; Cowman, Parravicini, Kulbicki, & Floeter, 2017; Kulbicki et al., 2013). Collectively, these locations encompass approximately 45% of all currently described coral reef fish species. To assess the taxa present in these ecoregions, we surveyed multiple species identification guides, including published ID books, as well as online databases like FishBase (www.fishbase.org) and Reef Life Survey (www.reeflifesurvey.com). After identifying which species have eyespots, fish images were sourced from the Smithsonian Institute's Division of Fishes Collections, supplemented by images from Williams et al. (2010). Images in this database or publication contain standard length (SL) and/or total length measurements (TL) which permit further measuring of morphological features (e.g., the eye). Furthermore, photographs of specimens in these collections have been photographed in a standardized manner, with the left side of the fish photographed, typically, shortly after death. However, not all individuals had their dorsal, anal, and caudal fins fully exposed which dictated whether the eyespot could be measured (see Figure 2). Therefore, this catalogue yielded two datasets which contained different information; each of which were used for different analyses. The first dataset contained length measurements of all eyespot‐bearing species in which the presence or absence of the marking could be identified (*n* species = 167, *n* samples = 1,140). For example, if the specimen photographed was in too poor of condition to allow for measurement (a consequence of preservation or the manner in which the individual was collected) but the presence/absence of an eyespot could be determined, its length and presence/absence was recorded. The second dataset contained only the images of specimens in high resolution of excellent preservation quality which permitted detailed measurements of the eyes and eyespot (*n* species = 140, *n* samples = 354). This dataset was used to make direct comparisons between the size of the eye and the size of the eyespot.

### 2.3 Morphological measurements and comparisons

We were curious if there is a distinct relationship between the size of the eyespot and the size of the eye. To test this, we measured four different features on both the eye and the eyespot. These four measurements were as follows: (1) the maximum diameter of the eye, (2) the maximum diameter of the pupil, (3) the area of the eye, and (4) the area of the pupil (Figure 2, further detail in Figure S3). These four features were measured using ImageJ (Schneider, Rasband, & Eliceiri, 2012) and were repeated on the matching features of the eyespot (e.g., the maximum width of the eyespots pupil). Each matched feature between the eye and eyespot was then compared using phylogenetic generalized least squares (PGLS) regression analyses due to the nonindependent nature of species data (the details of the phylogenetic tree used herein are described in the following section). Since the linear and area measurements measure the same features in alternative ways, only the area PGLS regression results are presented in the main text (linear regression results were identical and are presented in the Figure S2 and Table S1). In all analyses, we used the measurements of the eye as the explanatory variable and the measurements of the eyespot as the response variable. For species with more than one eyespot, measurements were taken of the largest eyespot. All measurements were converted into millimeters (mm) or mm^2^.

### 2.4 Phylogenetic backbone construction

To account for nonindependence due to shared evolutionary history (Felsenstein, 1985), a topology of all species included in this study was constructed using the most recent and comprehensive published phylogeny of coral reef fishes (Rabosky et al., 2018). Missing species were inserted based on previously published phylogenetic hypotheses (Betancur‐R et al., 2017). Replicate tips for each species were added to the tree depending on the number of images with measurable features available for each species. For example, five images of *Chrysiptera biocellata* were available that permitted measurements of the eye and eyespot. Therefore, four additional tips (since one tip was already present during the initial construction of the phylogeny) were added to the tree. These replicated tips have a branch length of zero, meaning each of the individuals within a species (e.g., *C. biocellata* #2 and *C. biocellata #*5) has the same phylogenetic distance to all other species. In doing this, we could add as many replicates for individuals within a species as there were images available, without letting species with many images drive relationships. This backbone was then incorporated into phylogenetic generalized least squares (PGLS) regression analysis to account for phylogenetic nonindependence (phylogenetic tree available in Figure S1).

### 2.5 Estimating size distributions and eyespot transitions

We utilized two separate statistical approaches to investigate the size at which fish have eyespots, and consequently, at what sizes this feature is lost. The first dataset (containing only eyespot presence/absence and standard length, *n* = 1,140) was divided into those fish with or without an eyespot. This yielded two separate datasets: one containing the size measurements of fish with eyespots (*n* species = 140, *n* samples = 586; 51.4%), and one containing the sizes of fishes that had or will have eyespots (*n* species = 115, *n* samples = 554, 48.6%). This is essentially the ecosystem perspective on eyespots: regardless of phylogeny, that is, at what size do fishes have eyespots and at what size do they not? To generate the size distribution of eyespot‐bearing and eyespot‐lacking individuals, a bootstrapping procedure was utilized that sampled one size measurement per species. This approach was used to account for variation in image sample‐sizes among species and more importantly, the variation in the size of individuals at which eyespots are lost or gained, that is, the size range where species may be transitioning from having an eyespot to losing it, or vice versa. This bootstrapping procedure is important since eyespots are gained or loss during ontogeny, but the size at which this happens differs for each individual within a species. These distributions were compared using a generalized linear model incorporating a gamma distribution which best accommodates the error structure of the data. The explanatory variable was eyespot presence or absence, and the response variable was the standard length (SL). This test was run for each iteration of the resampled dataset (250 times). Essentially, this approach tests if the size of individuals with eyespots is significantly different to those without.

This dataset can also be used to ask the question “what is the probability that a fish has an eyespot at a given size”? To answer this, we modeled the probability of possessing an eyespot at various standard lengths. This was done by using a binomial regression in which eyespot presence and absence were modeled as 1 or 0 (the response variable) and regressed against standard length (the explanatory variable). This was also run for 250 iterations. Additionally, the 50–50 point was calculated for each iteration. This is defined as the size in which the probability of having an eyespot is equal to the probability of not having it. All statistical analyses were conducted using the “stats” and “nlme” packages in R (Pinheiro, DebRoy, & Sarkar, 2019; R Core Team, 2019).

## 3 RESULTS

### 3.1 Morphological relationships

**FIGURE 3:**
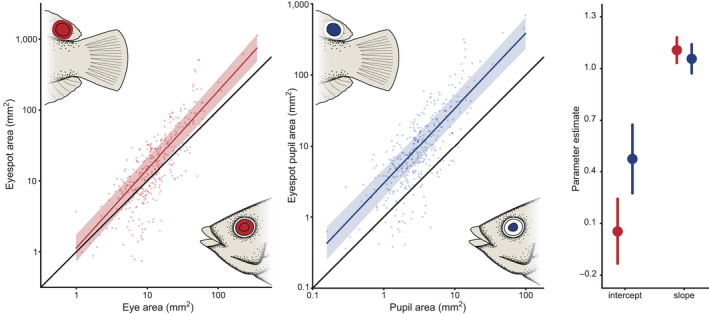
Relationship between the eye area and eyespot area (left) and pupil area and the eyespot “pupil” area (middle). The colored lines represent the phylogenetic generalized least squares regression (PGLS) lines with 95% confidence intervals. The solid black line represents an isometric growth pattern, where the area of one feature (in this case, the eye) would be equivalent to the area of the other feature (the eyespot). Estimates and 95% confidence intervals (right) of the regression models slope and intercepts for eye/eyespot (red) and pupil/eyespot pupil (blue). Both comparisons have identical slopes but different intercepts indicating that these features scale in the same manner; however, the eyespots pupil is consistently larger

The eyespot's total area and the eyespot's “pupil” area are significantly related to the area of the real eye and real pupil, respectively (Figure 3; all summary statistics in Table S2). The eyespot and the eye were almost identical in area. The slope was significant, indicating that the larger the individuals eye size, the greater in size of the eyespot. The slope was significantly greater than 1 which was likely driven by a few outliers of individuals which had the largest eyes recorded. However, since this relationship did not have an intercept significantly different than zero it indicates a consistent scaling between the size of the eyespot and the size of the real eye (Figure 3). Essentially, the size of the eyespot is almost identical to that of the real eye.

The real pupil area and the eyespot's “pupil” area were also significantly related with a slope that is not significantly different than 1. However, this relationship's intercept was significantly greater than zero and the confidence intervals were completely separated from the isometric line. In this case, the eyespot's “pupil” is consistently four times larger, on average, than the real pupil. When we compare the slope and intercept estimates from both models (the eyespot to eye and the eyespot pupil to the real pupil), it is evident that the slopes are not significantly different, but the intercepts are (Figure 3). Thus, in fish of all sizes, the overall size of the eyespot is remarkably similar to that of the eye, but the size of the eyespot's pupil is about four times larger.

### 3.2 Size distributions and eyespot loss

**FIGURE 4:**
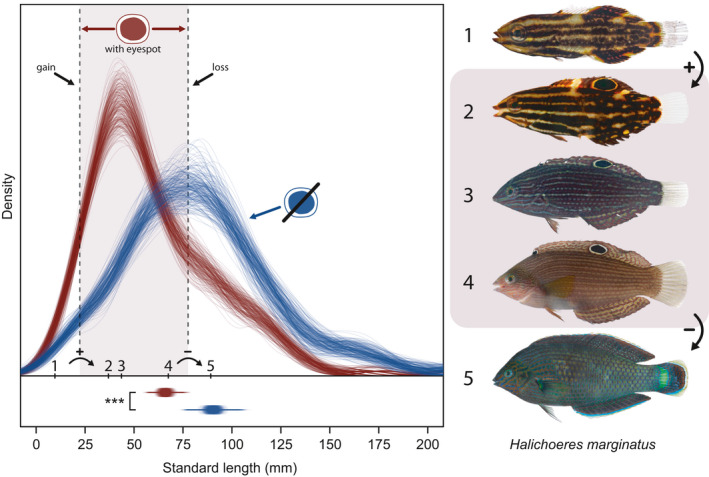
The size distribution of individuals with (red) and without (blue) an eyespot based on 250 bootstrapped size estimates. Below are the means and 95% confidence intervals plotted for each iteration. On the right are five individuals of *Halichoeres marginatus*, a species that displays the “window” of eyespot use in coral reef fishes. The number next to each image corresponds to their standard length measurement which are plotted on the main graph. Therefore, we can see the approximate sizes in which the eyespot is gained (+) and lost (−) marked by vertical dashed lines. Photographs: Jeffrey T. Williams, Smithsonian Institution

The size distributions of fishes with eyespots (*n* = 586; 51.4%) were significantly smaller than those without (*n* = 554, 48.6%; Figure 4). This pattern was consistent across all 250 iterations (median *p*‐value = .0007, mean *p*‐value = .002; histogram of all *p*‐values in Figure S4), indicating that even while accounting for individual variation, there were still pronounced differences. Individuals that possessed an eyespot were significantly smaller than the individuals of the same species which no longer had this marking. The variation within each iteration was low since most curves followed the same general trajectory; there was little influence of sampling differences among species.

**FIGURE 5:**
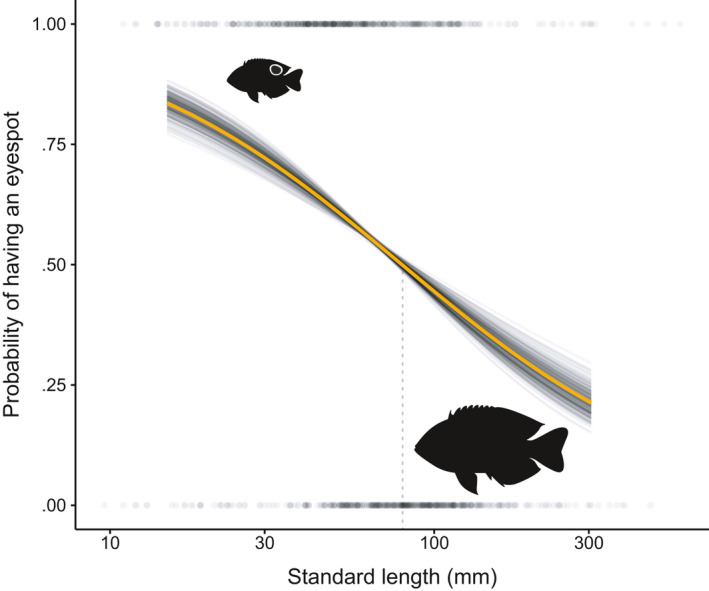
Probability of eyespot occurrence with increasing fish size. 250 bootstrapped binomial glms's displaying the relationship between standard length and the probability of having an eyespot. Each iteration's trendline is in black, the mean of these 250 trendlines is in orange. Additionally, the raw 1,140 specimen length measurements with their presence or absence (1 or 0, respectively) of an eyespot have been plotted

Furthermore, the standard length of an individual could significantly predict the probability of having an eyespot, with the highest probabilities occurring at the smallest sizes (Figure 5). All 250 iterations of the binomial glm were significant (both mean and median *p*‐value < .001; histogram of *p*‐values in Figure S5). The size in which a fish was equally likely to have/not have an eyespot (50–50 point) ranged from 75 mm to 85 mm (SL) depending on the iteration (mean: 79.77 mm, median: 79.51 mm; Figure S6). We can interpret this range of standard lengths as the sizes at which the costs of an eyespot outweigh the benefits.

## 4 DISCUSSION

### 4.1 Eye size induced constraints on eyespot size

The morphological regressions provide direct clues to the features that may determine eyespot size and structure in coral reef fishes. Since the area of the eyespot is approximately equivalent to the area of the eye at all fish sizes, eye size appears to strongly constrain the maximum effective size of eyespots. Essentially, it appears that the eyespot is not free to become as large as possible; it is strongly linked to eye size and presumably must be realistic. Such size constraints have been previously demonstrated in reef fishes, especially in ecological and morphological features (Mihalitsis & Bellwood, 2017; Schmitz & Wainwright, 2011b), but not with coloration. However, within this constraint, the eyespot's “pupil” is consistently and significantly larger than the real pupil. This suggests that the eyespot's pupil is responding to different selective pressures and thus may be responsible for drawing attention to this marking. Experimental studies are needed to explore this hypothesis and the extent to which pupil‐based conspicuousness plays a role in an eyespot's ability to grab attention (c.f. Kjernsmo, Grönholm, & Merilaita, 2018). Interestingly, the outer ring appears to be critically important in the anti‐predatory function of eyespots, as purely black, circular markings do not deter predators to the same extent (Winemiller, 1990). So, in reef fishes, eyespots do appear to resemble the overall size of the real eye, but the eyespot typically possesses an exaggerated pupil which may aid in grabbing the attention of predators.

Furthermore, the presence of eyespots appears to be an “all or nothing” phenomenon. In coral reef fishes, eye size displays negative allometry, that is, for a given increase in body size, the eye does not increase in size to a similar extent (Goatley & Bellwood, 2009; Howland, Merola, & Basarab, 2004; Schmitz & Wainwright, 2011a). Eyespots show similar, negative allometry (nonsignificantly different, Figure S7; Table S3), tracking an identical decrease in the relative eye size as body size increases. This suggests that to be effective eyespots must match eyes or be avoided entirely.

The notion of body size constraints on eyespot size has been found in caterpillars, butterflies, and freshwater fishes (Ho et al., 2016; Hossie et al., 2015; Kjernsmo et al., 2018; Kodandaramaiah, Lindenfors, & Tullberg, 2013). A lower limit based upon the size of the real eye makes ecological sense from a predation perspective. If attention is to be brought away from the real eye, logically, the eyespot would need to be realistic if not larger (Ho et al., 2016) (although eye concealment may mitigate this relationship (Kjernsmo, Grönholm, & Merilaita, 2016)). The upper limit remains more difficult to explain. Possibly, the upper limit reflects the size range of predators most likely to consume a prey of a given size. Restricting the upper limit may most effectively deter predators that are most likely to consume the individual (Kjernsmo & Merilaita, 2017).

### 4.2 Reduced presence with increasing size

The widespread and relatively rapid loss of an eyespot with growth suggests a marked decrease in their benefit during development. There appears to be a distinct “window” of eyespot effectiveness in coral reef fishes between 30 to 60 mm SL. Interestingly, a similar phenomenon has been described in caterpillars, with eyespots only benefitting species that are larger in size (Hossie et al., 2015). In this caterpillar example, eyespots were actually a detriment to smaller caterpillar species, increasing their probability of being attacked/eaten. A comparable scenario may operate in coral reef fishes. Many small coral reef fishes in the families Gobiidae and Tripterygiidae do not have eyespots although these families are particularly speciose. Furthermore, the majority of species within these families do not reach lengths greater than 50 mm, making them some of the smallest fishes on coral reefs (Brandl, Goatley, Bellwood, & Tornabene, 2018). They are therefore of a size that is subjected to some of the highest predation rates on reefs (Goatley & Bellwood, 2016). In this case, having purely cryptic coloration probably provides the most effective solution to avoiding predation at these smaller sizes (Cortesi et al., 2016; Lyytinen, Brakefield, Lindström, & Mappes, 2004). An eyespot would presumably draw the attention of predators, as in the caterpillar experiment.

The widespread loss of eyespots at 50–85 mm SL in multiple phylogenetically distinct lineages of reef fishes suggests that this loss is driven by strong selective pressure (Stevens, 2005). Of the samples surveyed herein, only 3.58% of individuals possessed this marking above 150 mm SL. Interestingly, the size at which fishes start to lose eyespots coincides with a significant decrease in the mortality rates of fishes on coral reefs. Previous research has shown that 43 mm total length is an important transition point in mortality rates for fishes on coral reefs (Goatley & Bellwood, 2016). Above this size threshold, predation pressure decreases substantially. The presence of an eyespot appears to reflect this threshold since eyespot presence decreases rapidly past this critical size. This narrow size window of eyespot presence (between 30 and 60 mm SL) offers support for the suggestion that eyespots in fishes may be functioning primarily as anti‐predatory mechanisms, among other mechanisms (Gagliano & Depczynski, 2013).

The consistent eyespot loss through development strongly suggests that their presence (and consequently, function) is strictly size‐dependent. If there were no costs in maintaining eyespots, they would in theory persist throughout the lifetime of many species. Since this is clearly not the case, there must be consequences for maintaining this feature through adulthood. Transitioning from prey to predator may operate in some species, that is, increasing crypsis as an adult. However, many reef fish species with eyespots as juveniles are not piscivorous as adults (e.g., all damselfishes and butterflyfishes; many wrasses). Clearly, there are strong selective pressures that constrain the presence of eyespots to moderately sized individuals. Investigating the fitness costs of eyespots for fishes offers a promising future avenue of research.

Herein, we identify the factors that determine eyespots size and form in coral reef fishes (matching eye size and maximizing the pupil) as well as evidence supporting a threshold associated with body size (the systematic acquisition then loss of eyespots through ontogeny). We show that eyespots have constraints that dictate how large they can become as well as the size of fishes that can utilize these markings. Our data highlight how certain factors can shape the appearance of an animal's coloration, since an eyespot is clearly for small, not large, reef fishes.

## CONFLICT OF INTEREST

None declared.

## AUTHOR CONTRIBUTIONS

**Christopher R. Hemingson:** Conceptualization (equal); data curation (equal); formal analysis (equal); investigation (equal); methodology (equal); validation (equal); visualization (equal); writing – original draft (equal); writing – review & editing (equal). **Peter F. Cowman:** Project administration (equal); supervision (equal); validation (equal); writing – review & editing (equal). **David R. Bellwood:** Conceptualization (equal); funding acquisition (equal); investigation (equal); project administration (equal); supervision (equal); validation (equal); writing – review & editing (equal).

## Supporting information

Supplementary Material

## Data Availability

Data available at: https://doi.org/10.25903/5ee057a8fe422.
