# Supplementary material for: Body size determines eyespot size and presence in coral reef fishes
Source: Ecol Evol. 2020 Jul 11;10(15):8144–52. doi: 10.1002/ece3.6509 (PMC7417216; doi:10.1002/ece3.6509)
Supplement: Supplementary file 1 — Supplementary Material [file ECE3-10-8144-s001.docx]

**Body size determines eyespot size and presence in coral reef fishes**

Christopher R. Hemingson^1,2,3^, Peter F. Cowman^3^, David R. Bellwood^1,2,3^

^1^ College of Science and Engineering, James Cook University, Townsville 4811, Australia

^2^ Research Hub for Coral Reef Ecosystem Function, James Cook University, 4811, Australia

^3^ ARC Centre of Excellence for Coral Reef Studies, James Cook University, 4811, Australia


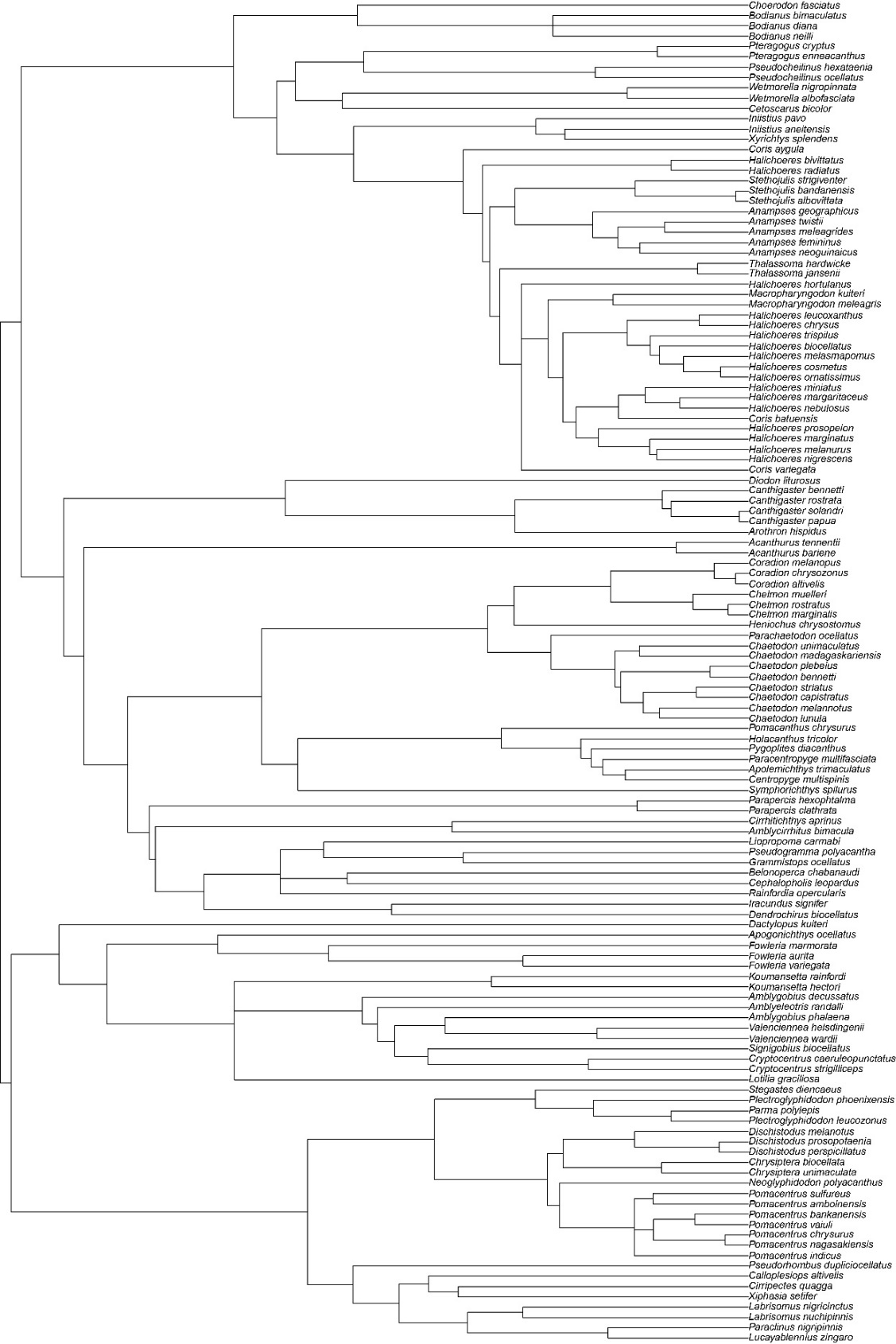


**Figure S1.** Phylogenetic tree of all species measured herein to account for non-independence in the regression analysis.


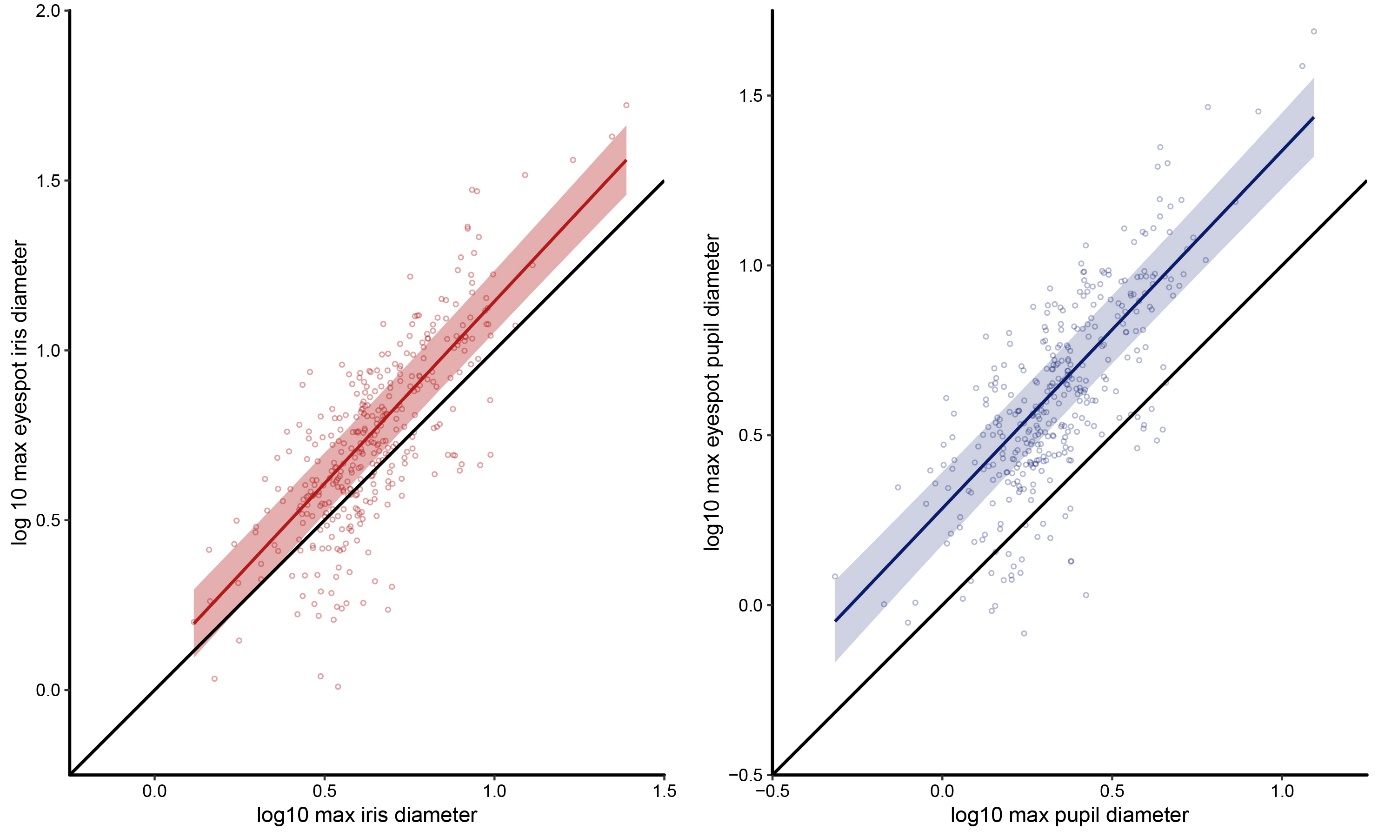


**Figure S2.** Morphological regressions using linear measurements of eye/eyespot (left) and pupil/eyespot pupil (right) features.

**Table S1.** Summary statistics from phylogenetic generalised least squares regression analysis of eye/eyespot diameter and pupil/eyespot ‘pupil’ diameter.

| *Predictors* | *Estimate* | *Confidence Interval* | *t - value* | *p - value* | *R^2^* |
| --- | --- | --- | --- | --- | --- |
| Intercept | 0.07 | -0.03 – 0.18 | 1.35 | 0.178 | 0.57 |
| log_10_(Eye Diameter) | 1.07 | 0.99 – 1.15 | 26.56 | **<0.001** |  |
| Intercept | 0.28 | 0.18 – 0.39 | 5.23 | **<0.001** | 0.54 |
| log_10_(Pupil Diameter) | 1.05 | 0.96 – 1.14 | 23.36 | **<0.001** |  |


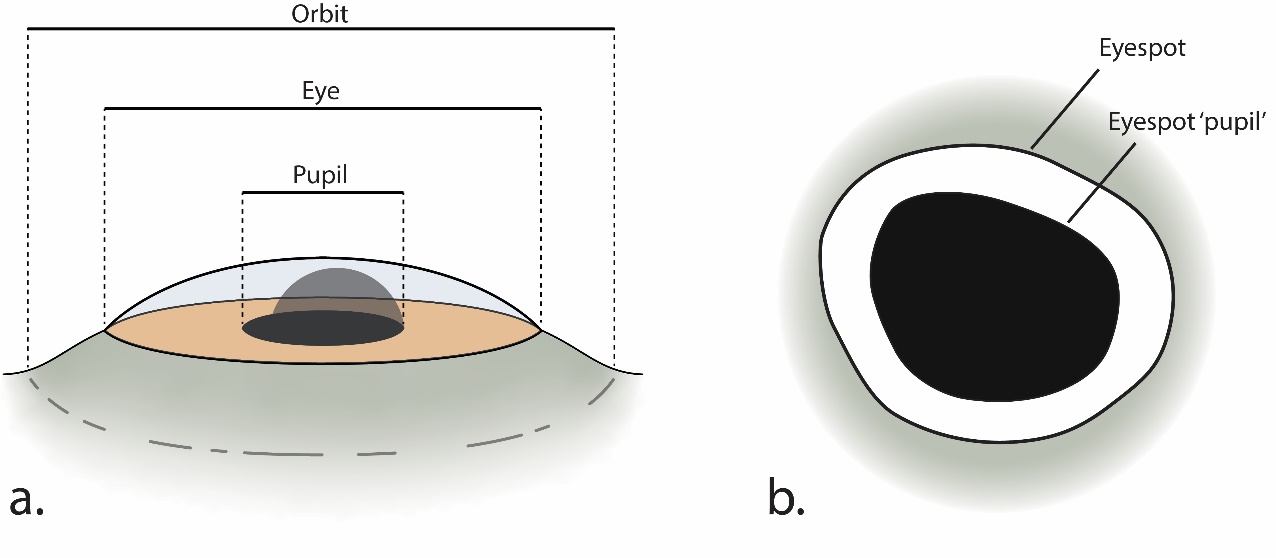


**Figure S3.** **a)** Lateral view of the eye’s external components. Herein, ‘eye area’ is defined to be the approximately flat disc that equivalates the iris in many other vertebrates (the orange disc in this figure). This feature is the outermost portion of the internal bones that form the sclerotic ring. **b)** The eyespots components. The eyespot’s area is the full area of the entire feature whereas the eyespots ‘pupil’ area is only that of the dark interior.


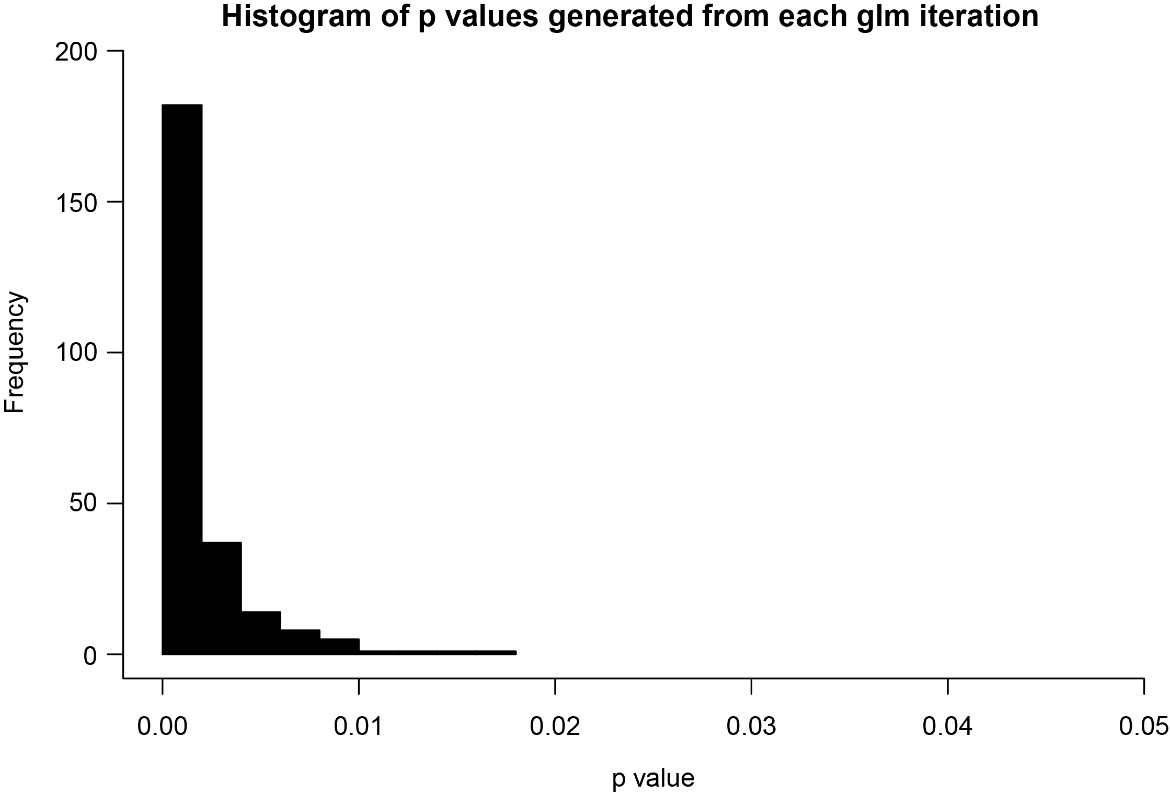


**Figure S4.** Distribution of p-values from the generalised linear model using a gamma distribution. This analysis was used to test for differences between the size distribution of fishes with and fishes that no longer/are yet to have an eyespot.

**Table S2.** Summary statistics from phylogenetic generalised least squares regression analysis of eye/eyespot area and pupil/eyespot ‘pupil’ area.

| *Predictors* | *Estimate* | *Confidence Interval* | *t - value* | *p - value* | *R^2^* |
| --- | --- | --- | --- | --- | --- |
| Intercept | 0.06 | -0.13 – 0.24 | 0.58 | 0.565 | 0.61 |
| log_10_(Eye Area) | 1.11 | 1.03 – 1.18 | 29.14 | **<0.001** |  |
| Intercept | 0.48 | 0.28 – 0.67 | 4.70 | **<0.001** | 0.55 |
| log_10_(Pupil Area) | 1.06 | 0.97 – 1.14 | 24.53 | **<0.001** |  |


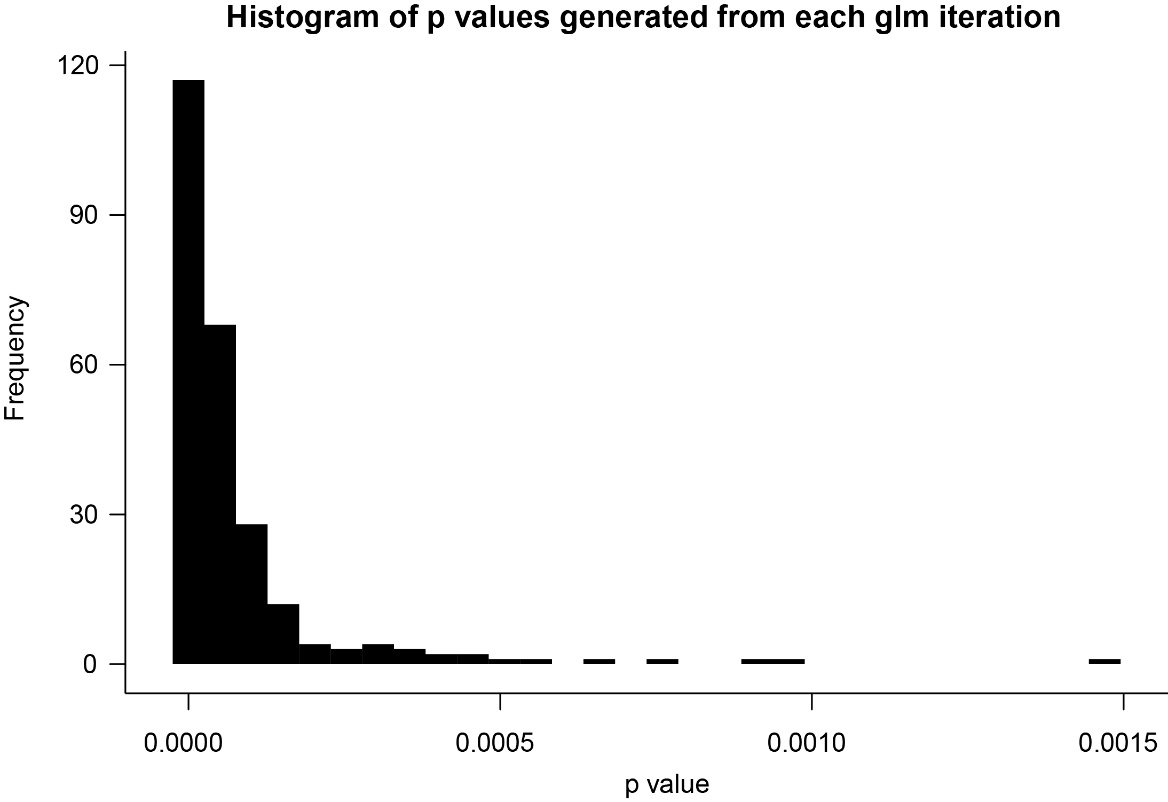


**Figure S5.** Distribution of p-values from the generalised linear model using a binomial distribution. This analysis was used to test for the relationship between standard length and the probability of having an eyespot. All 250 iterations were significant.


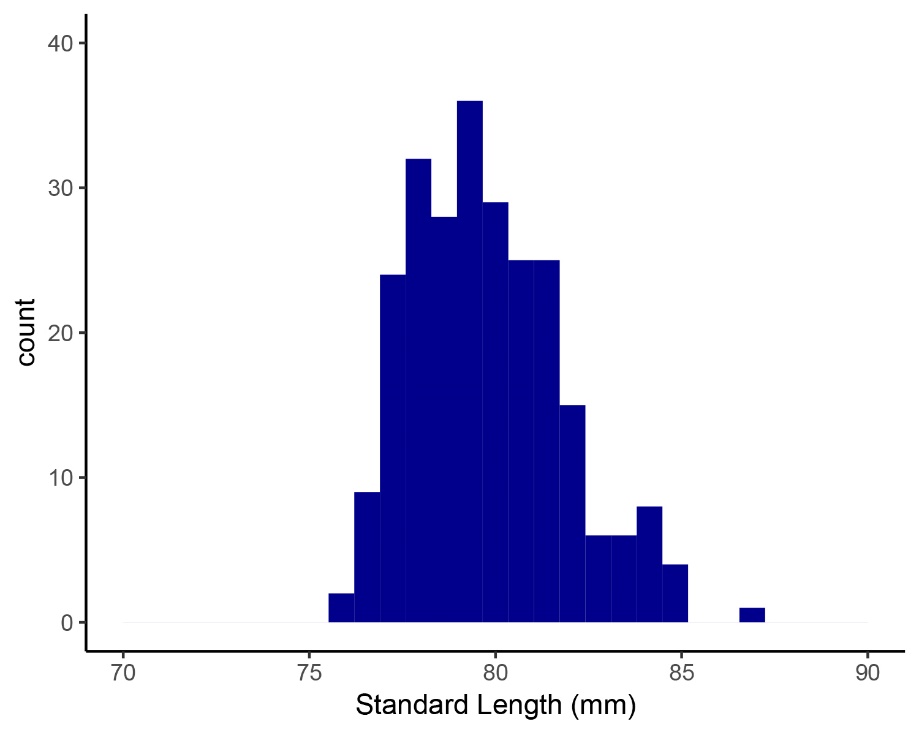


**Figure S6.** The distribution of 50-50 probability points for each of the 250 iterations of the binomial glm.


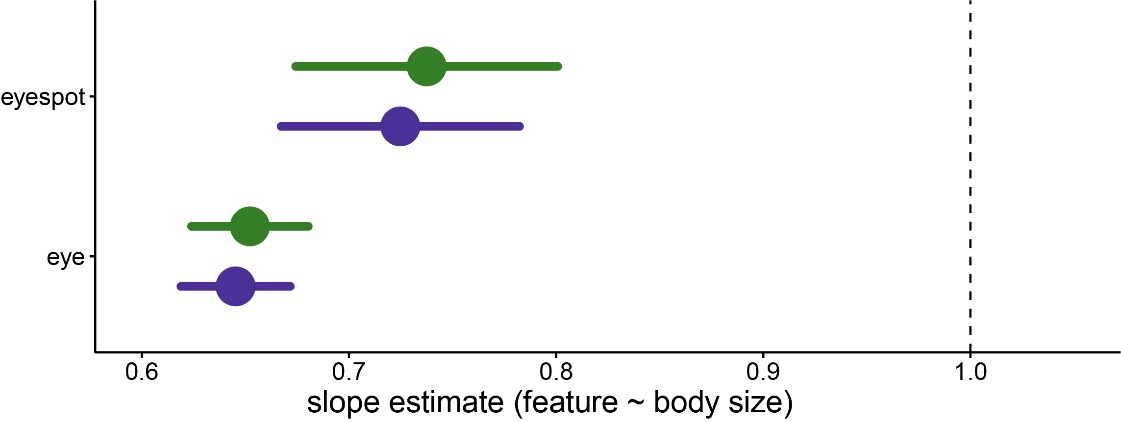


**Figure S7.** Slope estimates between body size and eye/eyespot size. Both body size and eye/eyespot diameters were log_10_ transformed to yield these slope estimates. Green indicates the max diameter of the full eye/eyespot; purple indicates the max diameter of the pupil/eyespot pupil. All slope estimates are <1 indicating negative allometry.

**Table S3.** Summary statistics from phylogenetic generalised least squares regression analysis of eye diameter, eyespot diameter, pupil diameter, and eyespot ‘pupil’ diameter. The explanatory variable in all models was the standard length (mm).

| *Response* | *Estimate* | *Confidence Interval* | *t - value* | *p - value* | *R^2^* |
| --- | --- | --- | --- | --- | --- |
| Intercept | -0.52 | -0.59 – -0.45 | -15.45 | **<0.001** | 0.75 |
| log_10_(Eye diam) | 0.65 | 0.62 – 0.67 | 48.09 | **<0.001** |  |
| Intercept | -0.55 | -0.69 – -0.41 | -7.49 | **<0.001** | 0.45 |
| log_10_(Eyespot diam) | 0.72 | 0.67 – 0.78 | 24.79 | **<0.001** |  |
| Intercept | -0.83 | -0.90 – -0.76 | -22.99 | **<0.001** | 0.69 |
| log_10_(Pupil diam) | 0.65 | 0.62 – 0.68 | 45.54 | **<0.001** |  |
| Intercept | -0.68 | -0.84 – -0.53 | -8.48 | **<0.001** | 0.42 |
| log_10_(Eyespot pupil diam) | 0.74 | 0.67 – 0.80 | 22.94 | **<0.001** |  |
